# Serum symmetric dimethylarginine shows a relatively consistent long-term concentration in healthy dogs with a significant effect of increased body fat percentage

**DOI:** 10.1371/journal.pone.0247049

**Published:** 2021-02-17

**Authors:** Amber Hillaert, Daisy J. X. Liu, Sylvie Daminet, Bart J. G. Broeckx, Emmelie Stock, Dominique Paepe, Myriam Hesta, Katrien Vanderperren

**Affiliations:** 1 Department of Medical Imaging of Domestic Animals and Orthopedics of Small Animals, Faculty of Veterinary Medicine, Ghent University, Merelbeke, Belgium; 2 Small Animal Department, Faculty of Veterinary Medicine, Ghent University, Merelbeke, Belgium; 3 Department of Nutrition, Genetics and Ethology, Faculty of Veterinary Medicine, Ghent University, Merelbeke, Belgium; University of Lincoln, UNITED KINGDOM

## Abstract

Symmetric dimethylarginine (SDMA) is a promising renal marker that correlates well with the glomerular filtration rate and could allow earlier detection of impaired renal function. The main objectives of this study were to assess the long-term variability of SDMA in healthy dogs and examine the influence of an increased body fat percentage on the level of SDMA. Sixteen lean Beagles were randomly assigned to the control group or weight-change group in age- and gender-matched pairs. The energy intake of the control group (n = 8) was strictly regulated to maintain an ideal body weight for 83 weeks, while the weight-change group (n = 8) was fed to induce weight gain (week 0–47), to maintain stable excessive body weight (week 47–56) and to lose weight (week 56–83), consecutively. At 8 specified time points, the body condition score, body composition, glomerular filtration rate, serum concentration of SDMA and creatinine were analyzed. In the control group, the within-subject coefficient of variation, between-subject coefficient of variation, reference change value (type I error = 5%) and index of individuality were 0.16, 0.22, 0.43 and 0.73, respectively. The control group and weight-change group did not differ significantly in SDMA concentration. SDMA showed a significant negative association (coefficient = -0.07) with body fat percentage (p<0.01) in the weight-change group and a significant positive association (coefficient = 7.79) with serum creatinine (p<0.01) in the entire study population. In conclusion, SDMA concentration has high long-term stability in healthy adult dogs. For the evaluation of SDMA concentrations, subject-specific reference values are preferred over a population-based reference value seen their higher sensitivity. Moreover, an increased body fat percentage does seem to affect the serum SDMA concentration of otherwise healthy dogs, but its clinical relevance has to be clarified in further research.

## Introduction

Symmetric dimethylarginine (SDMA) is a promising renal marker and has been proposed as an alternative to glomerular filtration rate (GFR) [1]. SDMA is released into circulation by eukaryotic cells following proteolysis and is mainly excreted by the kidney (≥90%) [2–5]. Studies in various species, such as humans, dogs and cats, have shown a good correlation between SDMA and GFR, and have demonstrated that SDMA provides added value in the detection and monitoring of impaired renal function [6–9]. SDMA is highly reliable as the number of confounding factors is limited [6, 10–12]. Unlike serum creatinine (sCr), SDMA is not influenced by age in adult dogs, lean body mass or gender [10–13]. Furthermore, SDMA might have advantages for the early detection of impaired renal function as 35 to 50% less nephron mass must be lost to cause a significant increase compared to sCr [6, 8, 9, 11, 13, 14].

To enable interpretation of SDMA results, a population-based reference-interval (6–13 μg/dl) and reference limit (<14 μg/dl) have been established based on healthy adult dogs [6, 15, 16]. However, data on SDMA variability, within and between healthy dogs, is necessary to assess the sensitivity of the population-based reference-interval to detect pathological changes in an individual over time [17, 18]. Nevertheless, this data is scarce and only known for relatively short periods [19]. Previously, Kopke et al. performed a variability study of two and a half months [19]. The researchers found a within-subject coefficient of variation (CV_I_) of 14% for SDMA, suggesting that the concentration remains consistent within this short timeframe [19].

So far, the influence of lean body mass on SDMA has been evaluated [9–11], while little data is available on the effect of obesity on the serum SDMA concentration in dogs. However, up to 20% of the canine population is affected by obesity, a chronic disease associated with excessive body fat accumulation [20–23]. In addition, data suggest that obesity might affect renal structure and function in both humans and dogs [24–29]. Experimental canine studies reported histological, hemodynamic and functional changes of the kidney during early weight-gain, including e.g. glomerular hypertrophy, increased mesangial matrix and thickening of glomerular and tubular basement membranes [27, 28]. Over time, chronic obesity may lead to progressive renal damage and development of chronic kidney disease [27–29]. Therefore, the renal function of obese dogs should be closely monitored [27–30]. Given the promising results, SDMA could play an important role in the follow-up of obese patients provided that the SDMA concentration is not affected by body fat percentage (BF%). In humans, studies show contradictory results on the effect of obesity on SDMA concentration [31–35].

The aims of this study were to assess the variability of SDMA in healthy dogs with stable kidney function over a longer period and to examine whether an increased BF% has an influence on the level of SDMA.

## Materials and methods

For this study, samples from a previously published longitudinal study of 1.5 years evaluating the effect of weight change on canine kidney function were used. For a detailed description of the study design and additional results, reference is made to the article by Liu et al. [27].

### Animals

This study included sixteen lean, purpose-bred research Beagles (eight male [four neutered] and eight female [two spayed]). At the start, the dogs were between 2.7 and 8.5 years old (mean ± standard deviation (SD), 4.4 ± 2.0 years), had a mean body weight (BW) of 11.4 kg (SD = 1.8 kg) and ideal body condition score (BCS) of 4-5/9 (mean ± SD, 4.1 ± 0.25) according to the 9-point scale of Laflamme [36]. All dogs were declared healthy based on a physical examination, comprehensive urinalysis and blood analysis. The Beagles were housed in controlled kennel conditions in accordance with European and national regulations for the care and use of animals at the Faculty of Veterinary Medicine (University of Ghent, Belgium). The study was approved by the Local Animal Ethics Committee of the Faculties of Veterinary Medicine and Bioscience Engineering, Ghent University (Project Number: EC2016/92). Following this research, the animals were housed for use in further studies.

### Experimental design

In the first four weeks of the study, the dogs were adapted to a commercially available adult maintenance dry diet (Veterinary™ HPM Adult Large and Medium, Virbac, Carros, France). Subsequently, the dogs were randomly assigned to the control group (CG) (n = 8) or weight-change group (WCG) (n = 8) in matched pairs based on age and gender.

For the dogs in the CG, their energy intake was strictly regulated to maintain an ideal BW (mean ± SD, 12.1 ± 1.7 kg) and BCS (mean ± SD, 4.1 ± 0.3) in all individuals for 83 weeks. The dogs in the WCG followed three consecutive phases, namely a weight gain phase (week 0–47), a weight stable phase (week 47–56) and weight-loss phase (week 56–83). The weight gain phase aimed to induce excessive BW or obesity, the weight stable phase aimed to maintain stable excessive BW, while the aim of the final phase was to return to the BW and BCS from the start of the study. The weight changes were induced by adjusting the energy intake by changing the amount of food from the same commercial diet.

Sample collection was performed at eight time points and started at week 0, 12, 24, 36, 47, 56, 68 and 83, respectively. For practical reasons, sampling was conducted over two weeks. On the second day of the first week, blood samples (12ml) were collected after a 12h fast and GFR was determined by plasma clearance of exo-iohexol. On the third day of the second week, morning urine samples in unsedated patients were collected by ultrasound-guided cystocentesis (22G needle). On the last day of the second week, body composition was determined by the deuterium oxide dilution method [37, 38]. An exception was made at week 68, when the measurements were performed within one week and were limited to blood sample collection and cystocentesis. At week 0, 24, 47, 56 and 83, a complete blood count and serum biochemistry profile (Architect C16000, Abbott Max-PlanckRing, Wiesbaden, Germany) were performed on all Beagles.

### Procedures

#### Body condition score and body composition

Based on visual inspection and palpation a BCS was assigned using a 9-grade scale [36]. Body composition was determined by the deuterium oxide dilution method using Fourier-transform infrared spectroscopy, as described by Ferrier et al. [37, 38]. Briefly, a blood sample was drawn from the jugular vein and collected in an EDTA tube after a 2h fast (water and food), followed by a subcutaneous injection of deuterium (500 mg/kg*)*. Four hours post-injection, a second sample was collected. These blood samples were spun at 2000 x g for 5 minutes at a temperature of 21°C as soon as possible. The plasma was stored in 300 μL Eppendorf tubes at -20°C, until further analysis of the deuterium concentration as described by Ferrier et al. [37].

#### Glomerular filtration rate

Clearance of exo-iohexol was used to estimate GFR, as previously described [39–41]. Briefly, blood was sampled from the jugular vein after 12-hour food deprivation, followed by an intravenous dose of iohexol (64.7 mg/kg BW, Omnipaque 300®, GE Healthcare, Diegem, Belgium). Post-injection, multiple blood samples were collected on pre-specified time points. Immediately after blood collection, the samples were centrifuged and the plasma was frozen in Eppendorf tubes at -20°C until assayed. The exo-iohexol concentration was measured by high-performance liquid chromatography (HPLC) [39, 41], after which a non-compartmental analysis (Phoenix 6.4, Princeton, NJ, USA) was performed to determine exo-iohexol clearance [41].

#### Laboratory variables

Serum SDMA concentrations were measured with a commercially available, novel, high-throughput, competitive homogeneous immunoassay (IDEXX SDMA^®^ Test, IDEXX Laboratories, Inc. Westbrook, ME 04092, United States) using a glucose-6-phosphate dehydrogenase conjugate and anti-SDMA monoclonal antibody [42].

As part of the serum biochemistry profile, sCr was determined by an automated chemistry analyzer (Architect C16000, Abbott Max-Planck-Ring, Wiesbaden, Germany). Concentrations lower than 1.4 mg/dl (123.76 μmol/l) were considered normal [43].

### Statistical analyses

The statistical analysis was conducted in R version 3.4.4. Significance was set at α ≤ 0.05. A random-effects model using restricted maximum likelihood (lme4 package) was used to estimate the variance components within the CG. Two variance components were estimated, v1 represents the variation between repeated measurements on the dog and v2 represents the extra variation when considering observations of different dogs. The variance components were used to determine 95% reference intervals for repeated observations in the same dog and for repeated observations in different dogs. The coefficient of variation (CV), defined as the ratio of the standard deviation over the mean, was determined for the within-dog repeated observations (CV_I_) and between-dog repeated observations (CV_G_). Furthermore, the reference change value (RCV) at a 5% confidence level and the index of individuality (IOI) were determined according to the ASVCP reference interval guidelines [44].

Next, the effect of changes in BF% on SDMA levels was analyzed in the WCG using a linear mixed model with dog as a random effect. Finally, in the total data set, four separate linear mixed models were used to evaluate whether the interaction between group (case/control) and time point was significant; SDMA, GFR, sCr or BF% were defined as dependent variables, group, time point and the interaction were defined as fixed effects, and dog was defined as a random effect. The association between SDMA (dependent variable) and GFR or sCr (fixed effect) was also assessed with a linear mixed model with dog as a random effect.

## Results

Results concerning BW, BCS and BF% were previously published by Liu et al. [27]. Briefly, the BF%, BW and BCS were significantly higher in the WCG from week 12 or 24 to week 56. At the end of the weight gain phase (week 47), three dogs of the WCG were overweight and five were obese.

**Fig 1:**
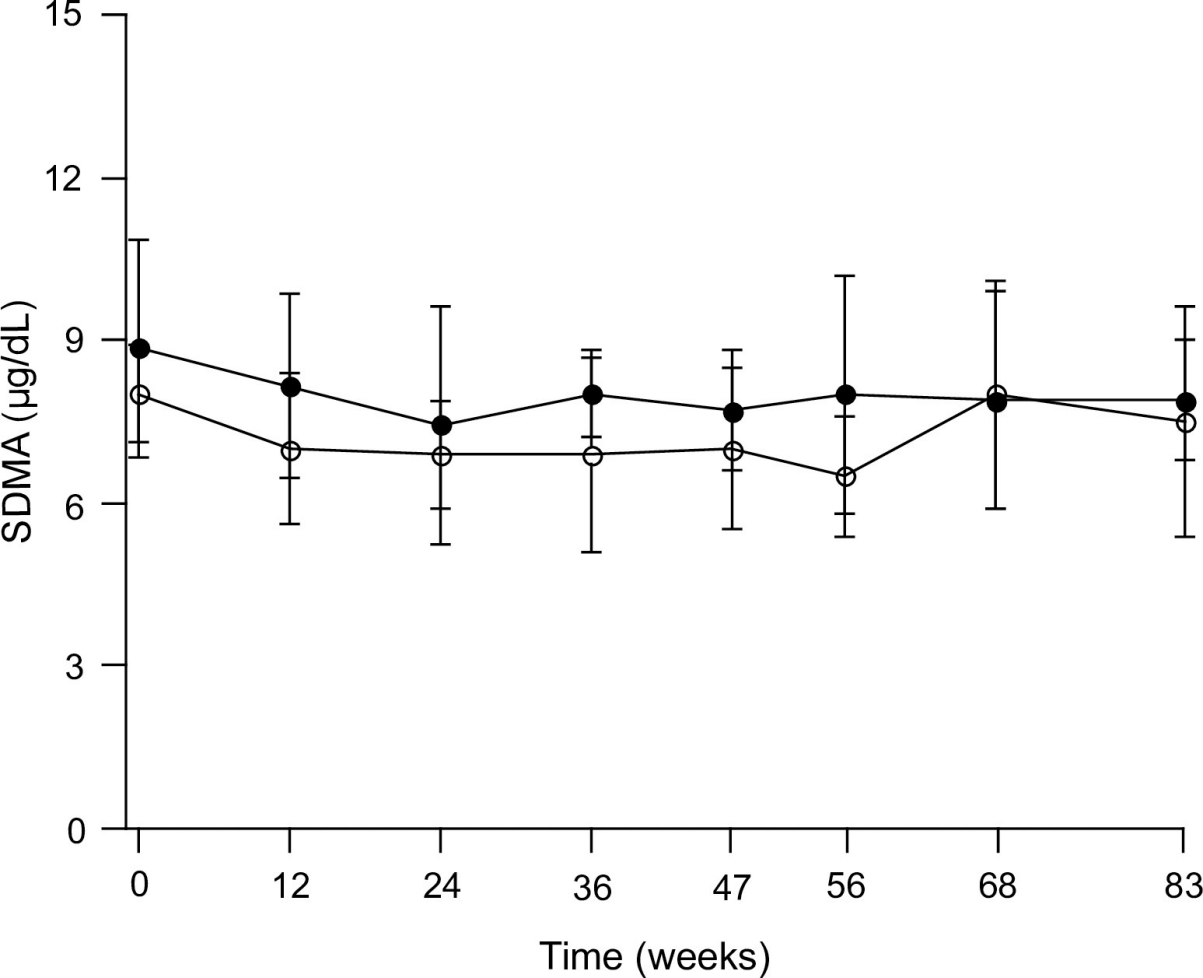
Serum SDMA concentration of the dogs in the control group (closed circles, n = 8)^a^ and weight-change group (open circles, n = 8) throughout the study. The weight-change group underwent three consecutive phases, namely the weight gain phase (week 0–47), weight-stable phase (week 47–56) and weight-loss phase (week 56–83). The subjects from the control group were kept at an ideal body weight. ^a^Data from 1 dog missing. SDMA, symmetric dimethylarginine.

Throughout the study, the serum SDMA concentration of all dogs remained below the upper reference limit (14 μg/dl). The lower reference limit of 6 μg/dl was crossed multiple times in both groups, with a minimal concentration of 4 μg/dl. In the CG, a 95% confidence interval within dogs (5.3–10.2 μg/dl) with a variance of 1.54, and a 95% confidence interval between dogs (4.3–11.2 μg/dl) with an added variance of 1.38 were established. The intercept (mean), standard error, CV_G_ and CV_I_ of SDMA in the CG were 7.76, 0.47, 0.22 and 0.16, respectively. Furthermore, an IOI of 0.73 and a RCV of 0.43 were calculated for SDMA. No significant difference was found over time between the CG and WCG. The results of the SDMA measurements over time are reported for both groups in Table 1. The course of the SDMA concentration for the CG and WCG during the weight-gain phase, weight-stable phase and weight-loss phase is visually represented in Fig 1.

**Table 1:** SDMA concentration (μg/dl) of the dogs in the Control Group (CG)^a^ and Weight-Change Group (WCG) over time.

Week		0	12	24	36	47	56	68	83
**CG**	Mean	8.86	8.14	7.43	8.0	7.71	8.0	7.88	7.88
	SD	2.0	1.7	2.2	0.8	1.1	2.2	2.0	1.1
**WCG**	Mean	8.0	7.0	6.88	6.88	7.0	6.5	8.0	7.5
	SD	0.9	1.4	1.0	1.8	1.5	1.1	2.1	2.1

The mean concentration and standard deviation (SD) at all measured time points are presented per group (n = 8). With dietary management, the control group was kept at an ideal body weight throughout the study (week 0–83). In the weight-change group, dietary adjustments were made to induce excessive weight gain (week 0–47), to maintain body weight (week 47–56) and to induce weight-loss (week 56–83), successively. ^a^Data from 1 dog missing. SDMA, symmetric dimethylarginine.

Results concerning sCr and GFR were also previously published by Liu et al. [27]. Briefly, sCr concentrations were within the laboratory reference interval for all dogs and was not significantly different between the CG and WCG over time. Similarly, no significant difference was detected for GFR between both groups. The profile of GFR from the two groups were parallel to each other, with slightly higher values in the CG. For both groups, a slight decrease could be observed until week 36. Afterwards, GFR of both groups showed a gradual increase until the end of the study.

**Fig 2:**
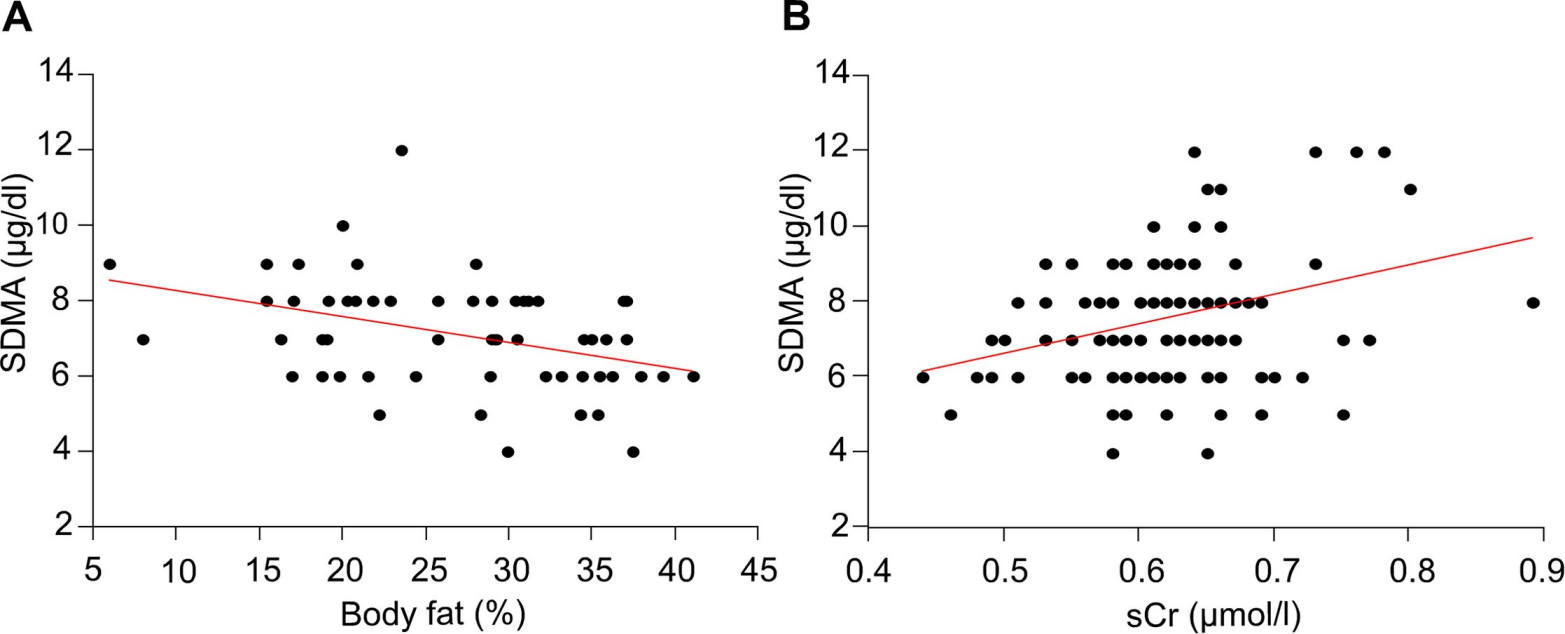
The association of SDMA with body fat percentage (A) and serum creatinine (sCr) (B) in dogs. SDMA showed a significant negative association (coefficient = -0.07) with body fat percentage (p<0.01) in the weight change group and a significant positive association (coefficient = 7.79) with serum creatinine (sCr) (p<0.01) in the entire study population^a^. ^a^Data from 1 dog missing in the control group. SDMA, symmetric dimethylarginine.

A significant association was found between SDMA and BF% (coefficient = -0.07, p<0.01) in the WCG and between SDMA and sCr (coefficient = 7.79, p<0.01) in the entire study population, respectively. SDMA and BF% are negatively associated, i.e., if the fat percentage increases by 1%, the SDMA concentration decreases by 0.07 μg/dl. In contrast, SDMA and sCr are positively associated, i.e., if sCr increases by 1 mg/dl, the SDMA concentration increases by 7.79 μg/dl. No significant association was found between SDMA and GFR. The association of BF% and sCr with SDMA is shown in Fig 2.

One of the control dogs was diagnosed with multicentric lymphoma in week 60 and was euthanized. The data from this dog were not included in the statistical analysis.

## Discussion

SDMA is a renal marker with growing importance in the early detection of renal dysfunction in dogs [1, 6–9]. Data on the variability of SDMA is necessary to assess the sensitivity of the population-based reference-interval in the detection of impaired renal function [17, 18]. Moreover, the renal function of obese dogs should be closely monitored, because of their increased risk on histological, hemodynamic and functional changes in the kidney [27–29]. However, prior research in dogs studied only the short-term variability of SDMA [19] and little data is available on the effect of obesity on the serum SDMA concentration in dogs, while a significant proportion of the pet dog population is overweight [20–22, 45, 46]. Therefore, the main objectives of this study were to assess the long-term variability of SDMA in healthy dogs and examine the influence of an increased BF% on the level of SDMA. In this long-term study of 1.5 years, SDMA showed a relatively high long-term stability in healthy adult dogs with an intermediate IOI. Moreover, a significant negative association was observed between SDMA and BF% in dogs with an excessive BW.

The within-subject coefficient of variation (CV_I_) indicates the extent to which serial measurements fluctuate within the same subject [47]. Knowledge of the variation of an analyte is essential as it can influence the interpretation of results [48]. This study found a relatively low long-term variability for SDMA, indicating that canine serum SDMA concentration is relatively consistent over long periods and that mild changes in serial measurements might be significant. Therefore, subject-specific reference values might be more sensitive in assessing relevant changes in SDMA than the population-based reference interval [49, 50]. Liu et al. previously studied the long-term within-dog variability of sCysC, uRBP, uNGAL, uIgG, and uCRP [51]. Compared to these renal biomarkers, except for sCysC (CV_I_ 8.1%), SDMA shows the lowest CV_I_.

Kopke et al. reported a similar CV_I_ of 14% for SDMA in a 10-week long study with an average sample interval of 8 days (range, 2–14 days) [19]. In contrast, the current study lasted more than 80 weeks, with an average sample interval of 12 weeks (range, 9–15 weeks). From these two studies, the sample interval appears to have a limited impact on the variation of SDMA. However, in general, a longer time interval between sample collection is thought to result in higher analytical and biological variability [49]. The minimal difference in CV_I_ between both studies is probably attributable to a small increase in biological variation over time as both studies applied the same assay and minimalized analytical variance by batch analyses of the samples at the end of the study. However, a substantial proportion of the within-subject variability is probably caused by the analytical error of the applied assay as a considerably high analytical variability ranging from 7.7% to 10% has been reported for the IDEXX SDMA test [19, 52, 53].

To determine whether the subject-specific reference values are preferable to a population-based reference interval, IOI can be used [49, 50]. When an analyte has a high individuality, i.e., low IOI (<0.6), subject-specific reference values are preferred as a measurement might fall within the population-based RI but is significantly different from an individual’s homeostatic set point [49, 54, 55]. When the IOI is larger than 1.4, subject-specific reference values add no additional information compared to a population-based RI and are no longer the method of first choice [49, 54, 55]. The intermediate IOI found in this study indicates that the population-based reference value may be used, but with caution as the population-based reference interval has limited sensitivity in this situation [49, 55]. Generally, the application of subject-specific reference values is often also recommended with an intermediate IOI [19]. The two main subject-specific reference values used for analyte evaluation are RCV and subject-based reference interval [49]. RCV refers to the percentage change that is minimally required to consider the difference between two consecutive results of an individual significant [56–58]. A subject-based reference interval, on the other hand, is a range of values that has been established based on measurements from one individual and is only applied to this same individual to evaluate subsequent results [49]. The RCV found in this study indicates that a difference of more than 43% between two consecutive SDMA measurements would be significant and cannot be ascribed to natural variation [18]. Previously, Kopke et al. found a similar IOI and RCV for SDMA of 0.87 and 47%, respectively [19]. A disadvantage of subject-specific reference values is that to be able to detect pathological changes, an individual’s normal value, based on serial measurements in an individual that is still healthy, has to be established first [19, 49].

Up to now, no major extra-renal influences on serum SDMA have been identified [6, 59]. However, the potential importance of SDMA as a diagnostic tool was only discovered recently, and the influence of many factors, such as obesity, is not yet well known [1, 60, 61]. Obesity is a pathology that might influence the variation around the set-point of SDMA or the set-point itself so that the evaluation of results using reference values based on healthy individuals could lead to misinterpretation [49, 62, 63]. A separate reference interval might be needed for obese dogs, as studies suggest that this group has a higher risk of developing renal dysfunction, and therefore monitoring these animals could be crucial [24–29]. In this study, SDMA was significantly associated with BF% in the WCG, indicating that there is a significant effect of increased BF% on the serum concentration of SDMA. However, no significant difference between the WCG and CG was found for SDMA despite a significantly higher BF%. Therefore, the clinical relevance of the negative association found between SDMA and BF% is unclear. The lack of a significant difference in SDMA concentration between both groups may be due to insufficient weight gain in the WCG or due to the limited power of this study to detect a significant difference. To exclude the latter, however, it needs to be determined which difference is considered clinically relevant. Further research needs to clarify whether an adjusted reference interval for obese dogs has to be established. In humans, various studies explored the relationship between SDMA and obesity [31–35]. However, their results are contradictory showing either no [34], a positive [31, 33], or a negative effect [32, 35] of obesity on SDMA.

The negative association found between SDMA and BF% contradicts the initial expectation. However, this result is in line with two studies in humans that reported a significantly lower SDMA concentration in juvenile and adult obese patients [32, 35]. The exact cause of this negative relationship is not clear, but researchers have proposed several theories that could be directly or indirectly linked with obesity [32, 64]. The first theory is that the negative association is the result of increased glomerular filtration and more efficient secretion of SDMA [32, 64]. Nevertheless, there were no direct indications of an enhanced renal function in studies that demonstrated a lower SDMA level in obese subjects [32, 35]. Previous studies do show, however, that obese individuals might be in a pre-diabetic or diabetic state associated with glomerular hyperfiltration which might result in the increased renal elimination of SDMA [65–67]. In this study, GFR was not significantly different between both groups and even showed a negative association with BF%. Two alternative theories that have been proposed for the negative relationship between SDMA and BW are increased cellular uptake and increased hepatic extraction of SDMA. Both mechanisms have been linked to elevated insulin levels associated with obesity-induced insulin resistance [64, 68].

To our knowledge, this study was the first to evaluate the long-term variability of serum SDMA in healthy dogs and to explore the effect of an increased BF% on the SDMA concentration while closely monitoring renal function by GFR. By including GFR measurements in the study design, possible changes in the SDMA concentration caused by renal dysfunction were excluded. Also, other potential sources of variability were kept to a minimum by the study design. Specifically, all laboratory dogs belonged to the same breed and were randomly divided into CG and WCG by age- and gender-matched pairs. They were all housed in a strictly controlled environment, received food with the same dietary composition, and samples were taken according to a predetermined protocol. Despite these strengths, the present study also had some limitations. The first limitation is the low number of subjects included in the study. As a result, a significant difference might not have been noticed. However, pre-analytical and analytical variations were minimized to avoid possible confounding factors that are not of interest and should allow the assessment of biological variation even with a low number of individuals [49, 55]. Complete elimination of the analytical variance was not possible, though, due to the difference in storage length between the first and last collected samples. The second limitation is the relatively high analytical variability of the applied SDMA assay, which has been reported to be between 7.7% and 10% [19, 52, 53]. According to published guidelines, the CV_A_ of an analytical method should be <0.5 CV_I_ to have a desirable performance [69]. In this study the CV_A_ fluctuates around the maximum acceptable value. Therefore, a potentially significant portion of the variance may be due to analytical imprecision. Future studies should include an extended study population of obese dogs and determine, in addition to SDMA, body composition, insulin level, insulin resistance and renal glomerular filtration rate.

In conclusion, SDMA concentrations in healthy adult dogs have relatively high long-term stability. Furthermore, extended sampling intervals seem to be associated with a minimal increase in biological variation. For the evaluation of SDMA concentrations, subject-specific reference values are preferred over a population-based reference value because of their higher sensitivity. Moreover, increased BF% does seem to affect the serum SDMA concentration of otherwise healthy dogs, but its clinical relevance has to be clarified in further research.
